# Incidental gallbladder cancer detected during laparoscopic cholecystectomy: conversion to extensive resection is a feasible choice

**DOI:** 10.3389/fsurg.2024.1418314

**Published:** 2024-09-05

**Authors:** Di Zeng, Yaoqun Wang, Ningyuan Wen, Jiong Lu, Bei Li, Nansheng Cheng

**Affiliations:** ^1^ Division of Biliary Tract Surgery, Department of General Surgery, West China Hospital, Sichuan University, Chengdu, Sichuan, China; ^2^ Research Center for Biliary Diseases, West China Hospital, Sichuan University, Chengdu, Sichuan, China

**Keywords:** incidental gallbladder carcinoma, hepatectomy, laparotomy, prognosis, propensity score analysis

## Abstract

**Background:**

Re-resection is recommended for patients with incidental gallbladder carcinoma (iGBC) at T1b stage and above. It is unclear whether continuation of laparoscopic re-resection (CLR) for patients with intraoperatively detected iGBC (IDiGBC) is more beneficial to short- and long-term clinical outcomes than with conversion to radical extensive-resection (RER).

**Methods:**

This single-centre, retrospective cohort study of patients with iGBC was conducted between June 2006 and August 2021. Patients who underwent immediate reresection for T1b or higher ID-iGBC were enrolled. Propensity score matching (PSM) was used to match the two groups (CLR and RER) of patients, and differences in clinical outcomes before and after matching were analyzed.

**Result:**

A total of 102 patients with ID-iGBC were included in this study. 58 patients underwent CLR, and 44 underwent RER. After 1:1 propensity score matching, 56 patients were matched to all baselines. Patients in the RER group had a lower total postoperative complication rate, lower pulmonary infection rate, and shorter operation time than those in the CLR group did. Kaplan-Meier analysis showed that the overall survival rate of patients who underwent CLR was significantly lower than that of patients who underwent RER. Multivariate analysis showed that CLR, advanced T stage, lymph node positivity, and the occurrence of postoperative ascites were adverse prognostic factors for the overall survival of patients.

**Conclusion:**

Patients with ID-iGBC who underwent RER had fewer perioperative complications and a better prognosis than those who underwent CLR. For patients with ID-iGBC, conversion to radical extensive-resection appears to be a better choice.

## Introduction

Gallbladder carcinoma (GBC) is the most common malignant tumor of the biliary tract and sixth most common malignancy of the gastrointestinal tract (1, 2). It is frequently diagnosed at an advanced stage because of its non-specific symptoms and aggressive biological behavior (3). Clinically, GBC can be divided into two categories based on the time of diagnosis. One is GBC clearly diagnosed before surgery, and the other is incidental gallbladder carcinoma (iGBC) discovered by pathological examination incidentally during or after surgery (4).

Approximately half of all patients diagnosed with GBC are detected incidentally during or after elective or emergency cholecystectomy (5). Epidemiological data suggest that the overall detection rate of incidental gallbladder cancer after routine cholecystectomy is approximately six per 1,000 (6). iGBC has a higher incidence in women than in men. At present, the age of onset of the disease also tends to be younger. In recent years, with the development and improvement of various auxiliary examination techniques, an increasing number of patients with GBC can be diagnosed preoperatively, which has prevented the occurrence of iGBC to a certain extent. However, with the increasing number of laparoscopic cholecystectomies performed, the clinical detection rate of iGBC is still increasing (7, 8).

The reported 5-year survival rates of patients with GBC after surgery varies widely, ranging from 10% to 100% (9–11). Indeed, long-term survival appears in patients with early-stage iGBC. Although systemic treatments such as chemotherapy (12–14), targeted therapy (15), and immunotherapy (16) have achieved remarkable results in biliary malignancies, radical resection is the most effective treatment for both resectable GBC patients and iGBC patients. Re-exploration and extensive-resection are currently recommended for patients with T1b, T2, and T3 iGBC without definite distant metastasis (17). For iGBC patients detected intraoperatively, immediate extensive-resection is necessary, and the ideal time for extensive-resection for the postoperative diagnosis of iGBC is 4–8 weeks after surgery (18). The extent of extensive-resection involves a partial hepatectomy of segments IVB + V, either as wedge resection or multi-segment resection and hepatoduodenal lymph nodes dissection (19). Recently, some studies have reported the results of laparoscopic extensive-resection in patients with iGBC (20, 21). However, most of these studies were based on iGBC detected postoperative. For intraoperatively detected iGBC (ID-iGBC), there is still a lack of research on whether to continue laparoscopic surgery or convert it to laparotomy.

In this study, to minimize bias due to lack of randomization, 1:1 propensity score matching (PSM) (22) was used for analysis. In patients with ID-iGBC, postoperative complications and prognoses were compared between the continuation of laparoscopic extensive-resection (CLR) group and conversion to laparotomy extensive-resection (COR) group. Ultimately, we will analyze and determine the optimal choice of extensive-resection, which may improve the prognosis of patients with ID-iGBC.

## Material and methods

### Inclusion and exclusion criteria

We retrospectively enrolled patients with ID-iGBC who underwent immediate extensive-resection at West China Hospital (Sichuan University, Chengdu, China) between June 2006 and August 2021.

Patients who met the following criteria were included: (1) male or female patients aged >18 years; (2) iGBC detected intraoperatively and confirmed by pathological examination; (3) patients who underwent radical extensive-resection (R0 and R1 resection); and (4) patient has no contraindication for hepatectomy. Patients meeting the following criteria were excluded: (1)T1a disease; (2) preoperative suspicion of GBC; (3) bile spillage during surgery; (4) history of any other primary malignancy except iGBC; (5) severe dysfunction of heart, kidney, or other vital organs.

### Basic characteristics assessment of patients

The preoperative assessment included the basic patient information and clinical laboratory indicators. The basic information of the patient mainly included sex, age, BMI, presence of diabetes, hypertension, coronary disease, history of stroke, and other medical history. Clinical laboratory indicators included (before extensive-resection): hemoglobin (Hb) (g/L), leukocyte (WBC) (10^9^/L), platelets (PLT) (10^9^/L), total bilirubin (TB) (mmol/L), serum albumin (ALB) (g/L), aspartate aminotransferase (AST) (IU/L) and alanine aminotransferase (ALT) (IU/L).

### Surgical technique

If a patient is diagnosedconfirmed with gallbladder cancer incidentally by intraoperative frozen-specimen examination, immediate extensive-resection should be performed. Whether extensive-resection is CLR or COR mainly depends on the the intraoperative decision of the surgeon team. The choice of surgical approach and the extent of liver resection were indeed influenced by the tumor stage and other factors such as tumor location and patient comorbidities. For T1b tumors, we generally performed a more extensive resection (e.g., IVb-V segmentectomy) compared to wedge resection, in accordance with current guidelines which recommend a more comprehensive resection for tumors with deeper invasion or those at higher risk of recurrence. This policy was uniformly applied regardless of the surgical approach (laparoscopic or open) to ensure oncological adequacy. To achieve negative resection margins, segment IVB + V resection and major hepatectomy were adopted (a major hepatic resection is currently defined as resection of three or more segments). Standard regional lymph node dissection was performed. The locations of regional lymph nodes were defined as follows: cystic duct, along the common bile duct, periduodenal, peripancreatic, portal vein and proper hepatic artery. To address potential bias from technological advancements, a stratified analysis of surgical outcomes was conducted based on the year of surgery and the implementation of laparoscopic technologies such as 3D imaging, 4 K resolution, and ICG fluorescence guidance. This approach aimed to assess whether these advancements influenced the comparative outcomes between laparoscopic continuation (CLR) and conversion to laparotomy extensive-resection (COR) groups.

### Pathological examination

During surgery, pathological examination was conducted using frozen section analysis of the gallbladder specimen. This method allows for rapid intraoperative diagnosis of gallbladder cancer, which is crucial for determining the extent of resection required.

### Postoperative strategy

Pathological evidence of cancer was determined using paraffin-embedded sections. All the included iGBC cases were histopathologically confirmed by an experienced pathologist. Hepatic invasion, choledochal invasion, and lymph node metastasis were pathologically examined. R0 resection was defined as the presence of a macroscopic and microscopic tumor-free resection margin. Complications were classified according to the Dindo-Clavien classification. Tumors were staged according to the American Joint Committee on Cancer (AJCC) classification (8th edition).

### Short-term outcomes

Surgery-related short-term clinical outcomes included total blood loss recorded in surgical records, blood transfusion (erythrocyte suspension or plasma), and duration of surgery. Liver function and routine blood examinations were performed on the 1st, 3rd, 5th, and 7th days after the operation to check for postoperative liver failure, jaundice, post-hepatectomy hemorrhage, or infection. For patients with infection symptoms, ultrasonography, chest CT and abdominal CT were used to further assess the cause of infection (pulmonary infection or abdominal infection). A daily physical examination was performed to check for biliary leakage, ascites, pleural effusion, and incision infection.

### Follow-up program and long-term outcomes

Within one year of discharge, the patients will be followed up every 3 months, and every six months after the first year. The follow-up mainly included blood routine blood tests, liver and kidney function, serum tumor markers, and medical imaging examinations (whole abdominal enhanced CT, MRI, etc.). The main clinical outcomes were overall survival (OS) and disease-free survival (DFS). OS was defined as the time from the end of surgery to death. DFS was defined as the time from the end of surgery to tumor recurrence.

### Statistical analysis

Patient data were retrospectively collected, and statistical analyses were performed using SPSS version 25.0 (SPSS Inc. Chicago, IL, USA). As we identified baseline characteristics mismatch between the two groups after patient grouping, we applied propensity score matching (PSM) analysis to minimize bias caused by non-randomized grouping. The variables selected for the propensity score model is listed in Table 1. Quantitative variables are expressed as mean (SD) if they presented a normal distribution, or otherwise as median and range. Qualitative variables are presented as absolute numbers and percentages. Normally distributed continuous data were compared using the student *t*-test and skewed-distributed by the Mann–Whitney *U*-test, and ordinal data were compared using a *χ*2 test or Fisher's exact test. Survival was described using the Kaplan–Meier method, and differences between subgroups were reviewed using the log-rank test. Mmultivariate analysis for prognostic factors was performed usingused a Cox proportional hazards model to analyze variables in univariate analyses with *P* < 0.05 in the univariate analyses. Two-sided *P* values <0.05 were considered to be statistically significant (23).

**Table 1:** Demographics and operative outcomes before and after matching.

Characteristics	Before matching (*N* = 102)	After matching (*N* = 56)
	No. of patients (%)	No. of patients (%)
Gender (F/M)	62/40	37/19
Age (years)	59.6 ± 12.2	60.6 ± 10.9
Over Weight (BMI >24)	40 (39.2%)	20 (35.7)
Diabetes	17 (16.7%)	7 (12.5%)
Hypertension	22 (21.6%)	13 (23.2%)
Stroke	3 (2.9%)	0 (0.00%)
Coronary heart disease	5 (4.9%)	2 (3.1%)
Hemoglobin(g/L)	117.4 ± 15.0	116.8 ± 15.8
WBC(109/L)	6.8 ± 2.8	6.8 ± 2.8
PLT(109/L)	158.6 (58.8–309.6)	153.6 (58.5–286.4)
AST(IU/L)	59.5 (17–379)	50.3 (17–208)
ALT(IU/L)	52.7 (10–322)	47.5 (10–235)
Obstructive jaundice	13 (12.7%)	4 (7.1%)
Hypoproteinemia	34 (33.3%)	18 (32.1%)
Differentiation		
Well	16 (15.7%)	8 (14.3%)
Moderate	72 (70.6%)	44 (78.6%)
Poor	14 (13.7%)	4 (7.1%)
Perineural invasion	20 (19.6%)	16 (28.6%)
Lymphovascular invasion	12 (11.8%)	6 (10.7%)
T stage		
T1b	15 (14.7%)	12 (21.4%)
T2	70 (68.6%)	33 (58.9%)
T3	17 (16.7%)	11 (19.6%)
N stage		
N0	87 (85.3%)	53 (94.6%)
N1	15 (14.7%)	3 (5.4%)
8th AJCC stage		
I	15 (14.7%)	12 (21.4%)
II	61 (59.8%)	33 (58.9%)
IIIA	11 (10.8%)	8 (14.3%)
IIIB	15 (14.7%)	3 (5.4%)
Intraoperative ultrasonography	65 (63.7%)	34 (60.7%)
Type of re-resection		
CLR	58 (56.9%)	28 (50.0%)
COR	44 (43.1%)	28 (50.0%)
Type of hepatectomy		
Segment IVB + V resection	79 (77.5%)	42 (75.0%)
Major hepatectomy	23 (22.5%)	14 (25.0%)

## Results

### Baseline characteristics

A total of 102 patients diagnosed with iGBC during laparoscopic cholecystectomy at our hospital between June 2006 and August 2021 were retrospectively evaluated. We divided the patients into two groups (44 and 58 in the COR and CLR subgroupsrespectively) according to the type of extensive-resection. Table 1 shows the demographics and operative outcomes of the patients with ID-iGBC before and after matching. The baseline characteristics in terms of hypoproteinemia, perineural invasion, and age showed significant or slight differences before matching. After 1:1 matching, 28 patients in the COR subgroup and 28 patients in the CLR subgroup were matched (caliper = 0.2), with all baselines balanced. Table 2 shows the baseline characteristics of the two groups before and after PSM.

**Table 2:** Baseline characteristics of patients in CLR group and COR group before and after PSM matching.

Characteristics	Before matching (*N* = 102)			After matching (*N* = 56)		
	CLR (*N* = 58)	COR (*N* = 44)	*P*-value	CLR (*N* = 28)	COR (*N* = 28)	*P*-value
Gender			0.917			0.778
Male	23	17		18	19	
Female	35	27		10	9	
Age (years)	58.1 ± 13.1	61.6 ± 10.7	0.053	61.5 ± 11.0	59.7 ± 11.0	0.754
Over Weight (BMI>24)	26	14	0.183	11	9	0.577
Diabetes	11	6	0.474	4	3	0.686
Hypertension	13	9	0.812	6	7	0.752
Stroke	3	0	0.126	0	0	1.000
Coronary heart disease	4	1	0.284	1	1	1.000
Hemoglobin(g/L)	117.4 ± 15.3	117.2 ± 14.9	0.947	118.4 ± 15.8	115.2 ± 15.8	0.516
WBC(10^9^/L)	6.6 ± 3.0	7.1 ± 2.6	0.332	6.9 ± 3.3	6.7 ± 2.4	0.129
PLT(10^9^/L)	154.45 (58.5–309.6)	137.3 (71.3–285.5)	0.253	151.95 (58.5–286.4)	137.3 (79.4–285.5)	0.928
AST(IU/L)	45 (19–379)	40 (17–235)	0.278	42.5 (19–208)	40 (17–125)	0.461
ALT(IU/L)	36 (11–322)	31.5 (10–230)	0.461	33.5 (11–235)	26.5 (10–230)	0.342
Obstructive jaundice	9	4	0.335	3	1	0.299
Hypoproteinemia	24	10	0.048*	9	9	1.000
Differentiation			0.998			0.580
Well	9	7		4	4	
Moderate	41	31		21	23	
Poor	8	6		3	1	
Perineural invasion	7	13	0.028*	7	9	0.554
Lymphovascular invasion	8	4	0.465	3	3	1.000
T stage			0.186			0.162
T1b	7	8		4	8	
T2	44	26		20	13	
T3	7	10		4	7	
N stage			0.163			0.533
N0	47	40		26	27	
N1	11	4		2	1	
8th AJCC stage			0.410			0.161
I	7	8		4	8	
II	35	26		20	13	
IIIA	5	6		2	6	
IIIB	11	4		2	1	
Intraoperative ultrasonography	38	27	0.666	17	17	1.000
Type of hepatectomy			0.659			0.537
Segment IVB + V resection	44	35		20	22	
Major hepatectomy	14	9		8	6	

### Short-term outcomes and long-term outcomes

Table 3 shows a comparison of differences in perioperative clinical outcomes (surgery-related outcomes and postoperative complications) before and after PSM. Surgery-related outcomes, such as R1 resection rate, volume of intraoperative hemorrhage, and intraoperative transfusion, were not significantly different between the two groups before and after PSM. Postoperative complications, such as total postoperative infection rate, incisional infection, sepsis, liver abscess, liver failure, ascites, pleural effusion, bile leakage, and postoperative hemorrhage, were not statistically significant between the two groups before and after matching. Significant differences were found in the operation time, total postoperative complications, and pulmonary infection between the two groups. The COR group had a shorter operation time than the CLR group [175 (90–330) vs. 255 (145–500) before PSM, *p* < 0.001; 177.5 (90–330) vs. 252.5 (145–375) after PSM, *p* = 0.001]. Total postoperative complications (20 vs. 7 before PSM, *p* = 0.035; 10 vs. 3 after PSM, *p* = 0.027) and pulmonary infections (10 vs. 2 before PSM, *p* = 0.049; 6 vs. 1 after PSM, *p* = 0.043) occurred significantly more frequently in those who underwent CLR than in those who underwent COR. Although patients in the CLR group before PSM had a longer hospital stay than those in the COR group [13 (7–48) vs. 10.5 (6–30) before PSM, *p* = 0.009], it was found after PSM that the length of hospital stay showed no statistically significant difference between the two groups [11.5 (7–48) vs. 10 (6–17) after PSM, *p* = 0.119].

**Table 3:** Short-term clinical outcomes of patients in CLR group and COR group and delayed re-resection group before and after PSM matching.

Characteristics	Before matching (*N* = 102)			After matching (*N* = 56)		
	CLR (*N* = 58)	COR (*N* = 44)	*P*-value	CLR (*N* = 28)	COR (*N* = 28)	*P*-value
Operation time (min)	255 (145–500)	175 (90–330)	<0.001*	252.5 (145–375)	177.5 (90–330)	0.001*
R1 resection	11	5	0.296	1	3	0.299
Intraoperative hemorrhage (ml)	400 (200–1,500)	380 (50–1,250)	0.065	400 (200–750)	340 (50–1,250)	0.153
Intraoperative transfusion	7	4	0.631	2	3	0.639
Intraoperative transfusion RBC (ml)	300 (200–800)	350 (200–600)	0.428	250 (200–300)	400 (200–600)	0.374
Suspensions (ml)	200 (0–600)	150 (100–400)	1.000	100 (0–200)	200 (100–400)	0.374
Total postoperative complications	20	7	0.035*	10	3	0.027*
Total postoperative infection	12	5	0.211	7	3	0.163
Pulmonary infection	10	2	0.049*	6	1	0.043*
Incisional infection	3	3	0.726	1	3	0.299
Sepsis	1	0	0.381	1	0	0.313
Liver abscess	1	1	0.843	0	0	1.000
Liver failure	1	0	0.381	0	0	1.000
Ascites	4	1	0.284	2	1	0.553
Pleural effusion	9	6	0.791	5	3	0.445
Bile Leakage	3	2	0.885	1	1	1.000
Postoperative hemorrhage	4	0	0.076	2	0	0.150
Hospital stay (day)	13 (7–48)	10.5 (6–30)	0.009*	11.5 (7–48)	10 (6–17)	0.119

Stratified analysis based on the year of surgery and technological advancements revealed that while newer laparoscopic technologies may enhance precision and outcomes, their impact on the comparative results between CLR and COR groups was not statistically significant in our study cohort (10). This finding suggests that the core advantages of laparoscopic surgery, such as reduced postoperative complications and shorter hospital stays, persisted across different technological eras.

### Prognosis factors in patients with ID-iGBC

**Figure 1:**
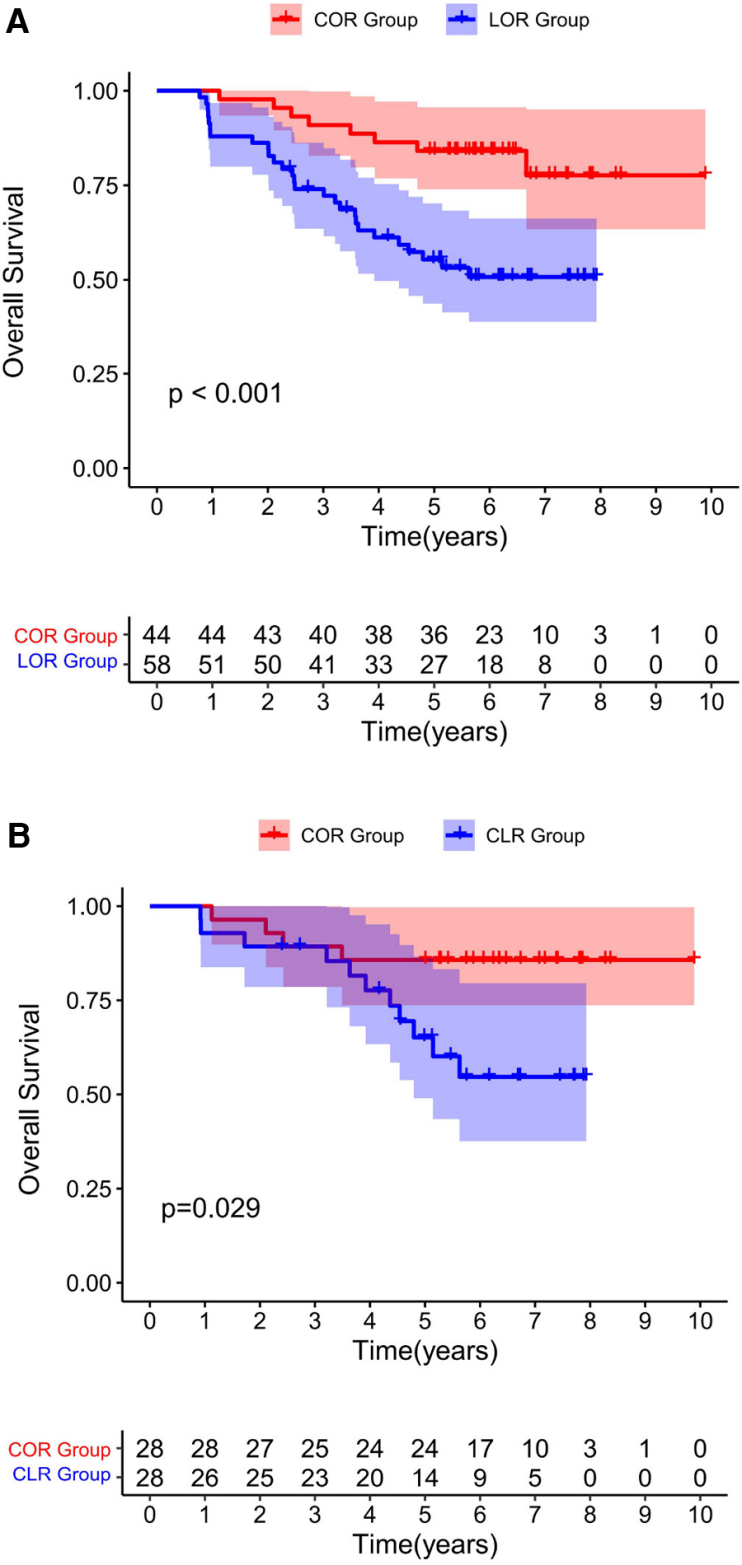
Overall survival for all patients underwent a re-resection. Before PSM **(1A)**. After PSM **(1B)**. Both before and after matching, the COR group was associated with improved OS compared to the CLR group.

**Figure 2:**
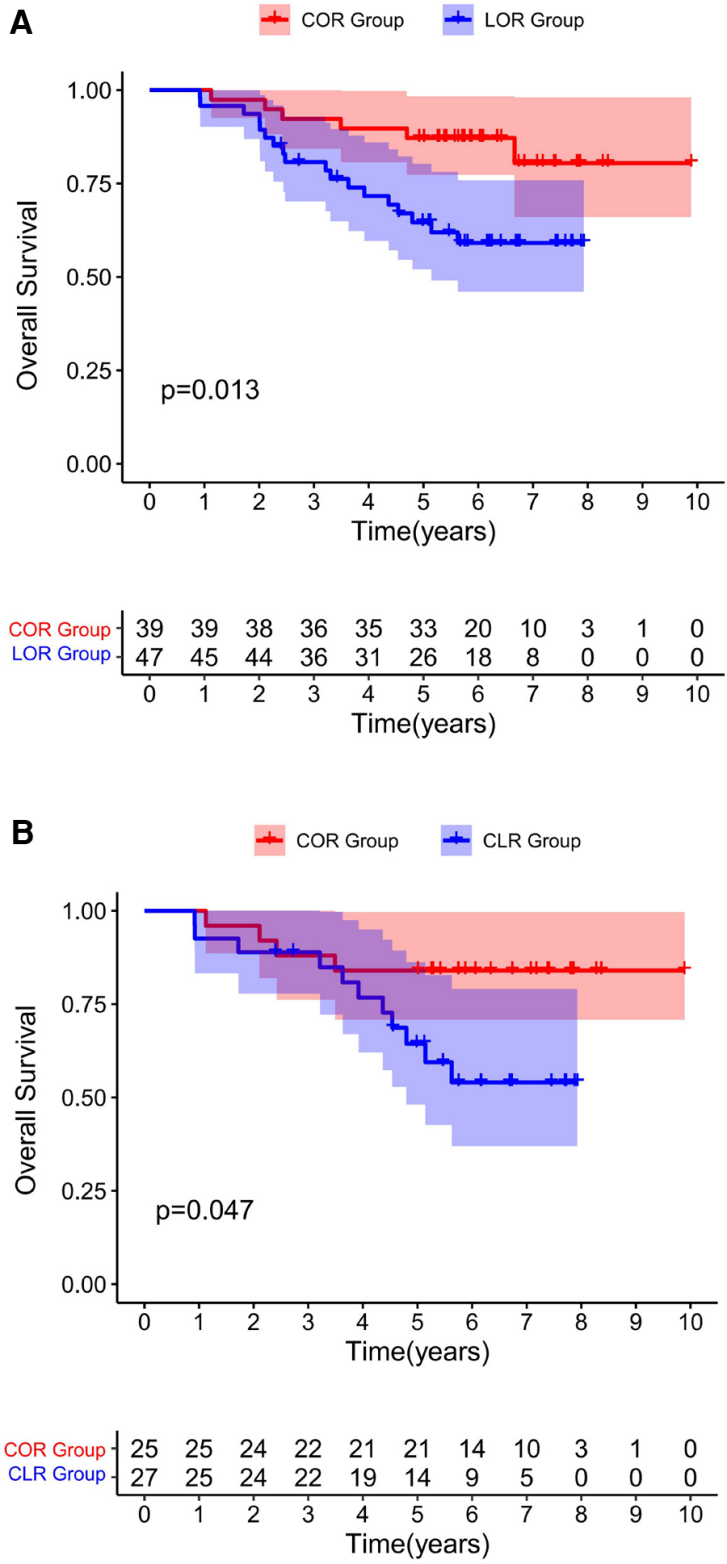
Overall survival for all patients underwent a re-resection, excluding R1 resections. Before PSM **(A)**. After PSM **(B)** Both before and after matching, the COR group was associated with improved OS compared to the CLR group.

The univariate and multivariate Cox proportional hazards analyses for OS calculated from the date of extensive-resection are shown in Tables 4, 5. The specific mortality data will be presented in Figures 1 and 2. COR [HR = 0.11(0.04–0.31), *p* < 0.001] and intraoperative ultrasonography [HR = 0.45(0.23–0.91), *p* = 0.026] were associated with better survival on pre-PSM multivariable analysis, advanced T stage [HR = 3.11(1.34–7.25), *p* = 0.008] and advanced 8th AJCC stage [HR = 4.11(2.49–6.80), *p* < 0.001] were associated with worse survival.

**Table 4:** Univariable and multivariable cox regression analysis for overall survival before PSM matching.

Variable	Univariate analysis HR (95% CI)	*P*-value	Multivariate analysis HR (95% CI)	*P*-value
COR	0.30 (0.13–0.66)	0.003*	0.11 (0.04–0.31)	<0.001*
Differentiation	2.45 (1.01–5.90)	0.046*		
T stage	42.05 (8.98–196.90)	<0.001*	3.11 (1.34–7.25)	0.008*
*N* stage	10.90 (5.13–23.19)	<0.001*		
8th AJCC stage	37.97 (13.11–109.95)	<0.001*	4.11 (2.49–6.80)	<0.001*
Intraoperative ultrasonography	0.55 (0.29–1.08)	0.082	0.45 (0.23–0.91)	0.026*
Major hepatectomy	1.82 (0.90–3.65)	0.094		
Operation time (min)	3.16 (1.47–6.81)	0.003*		
Intraoperative hemorrhage (ml)	1.58 (0.94–2.64)	0.081		
R1 resection	4.01 (1.94–8.30)	<0.001*		
Liver failure	9.59 (1.23–74.93)	0.031*		
Ascites	4.13 (1.44–11.80)	0.008*		

**Table 5:** Univariable and multivariable cox regression analysis for overall survival after PSM matching.

Variable	Univariate analysis HR (95% CI)	*p*-value	Multivariate analysis HR (95% CI)	*p*-value
COR	0.30 (0.09–0.94)	0.039*	0.09 (0.02–0.38)	0.001*
T stage	157.28 (14.47–1,709.86)	<0.001*	23.04 (5.44–97.56)	<0.001*
*N* stage	23.89 (5.56–102.56)	<0.001*	9.51 (1.31–69.12)	0.026*
8th AJCC stage	105.68 (15.58–716.98)	<0.001*		
Ascites	4.27 (0.96–19.03)	0.057	9.32 (1.25–69.73)	0.030*

After PSM, multivariable analysis showed that only COR [HR = 0.09(0.02–0.38), *p* = 0.001] was associated with better survival and advanced T stage [HR = 23.04(5.44–97.56), *p* < 0.001], advanced N stage [HR = 9.51(1.31–69.12), *p* = 0.026] and the occurrence of postoperative ascites [HR = 9.32(1.25–69.73), *p* = 0.030] were associated with poor survival.

## Discussion

GBC is a rare and aggressive malignancy with poor prognosis. Surgical resection is the only curative treatment (24, 25). Most guidelines recommend incidental discovery of Tis and T1a tumors in patients who underwent simple cholecystectomy alone (26–28). For patients with iGBC with T1b and higher tumors, radical extensive-resection is recommended, including resection of the adjacent liver parenchyma, involved organs, and complete regional lymphadenectomy to obtain negative margins (26, 29). The extensive-resection strategies may also be an important and heretofore underappreciated determinant of outcomes in patients with ID-iGBC. The optimal surgical strategy for immediate re-excision of ID-iGBC remains unknown. In recent years, researches related to iGBC have mainly focused on patients with iGBC found after surgery. The optimal timing of surgical resection (18, 30, 31), the safety of laparoscopic surgery (20, 21) and specific surgical approach for iGBC resection (32, 33) have been extensively studied in these articles. However, no study has focused on patients diagnosed with iGBC during laparoscopic cholecystectomy. For ID-IGBC, a relatively special group of patients, lacks sufficient preoperative preparation and detailed preoperative examination materials. Simultaneously, owing to the discovery of ID-iGBC, the surgical plan has changed from simple cholecystectomy to complex radical resection of gallbladder cancer, which is undoubtedly a huge challenge for both the patient and the attending physician team. To fill this gap in this research field, we conducted a single-center retrospective cohort study.

In present study, ID-iGBC patients were divided into COR and CLR subgroups according to the surgical approach. If the patient who were presumed benign gallbladder disease preoperatively and confirmed as gallbladder cancer incidentally by intraoperative frozen-specimen examination, immediate extensive-resection should be performed. Patients who continued to undergo laparoscopic radical resection of gallbladder cancer were included in the CLR group, and those who were converted to laparotomy were included in the COR group. If gallbladder cancer was confirmed only by postoperative paraffin section histopathological examination, the patient was excluded. Based on data from the current study, patients in COR group had a better long-term survival than those in COR group even when excluding patients with R1 resection after propensity score matching (Figure 2B). The possible reasons for this are many. First, T1b and higher iGBC may present residual disease (RD) after cholecystectomy alone (34). Second, since these patients are not fully prepared for liver resection before surgery, early conversion to laparotomy will help surgeons to fully detect liver invasion, make more accurate surgical resection plans, and obtain a higher R0 resection rate (35).

The COR group had shorter operation time, less total postoperative complications rate and less pulmonary infection rate compared with the CLR group. Gallbladder inflammation is present in most patients with ID-iGBC. Studies have pointed out that COR can better prevent the occurrence of serious complications such as biliary tract injury, bleeding, and ensure the safe completion of the operation. The conclusion of our study further confirmed this view. The time required to dissect adhesions, particularly in cases complicated by inflammation, remains a topic of debate regarding its impact on operative duration, especially between laparoscopic and open approaches. Further high-quality research is needed to provide robust evidence in this area (36, 37).

Interestingly, our study did not find a significant difference in postoperative length of stay between laparoscopic and open surgery. This may be attributed to several factors, including case complexity and the presence of postoperative complications that can negate the usual benefits of laparoscopic surgery. Additionally, variations in postoperative care protocols across institutions could influence length of stay. In our study, the longer hospital stays for laparoscopic surgeries (13 days) compared to open surgeries (10.5 days) likely stems from these factors, along with differences in discharge criteria and postoperative management strategies. Laparoscopic procedures, despite their usual benefits of reduced pain and faster recovery, can sometimes necessitate extended hospital stays due to factors like longer surgery times in complex cases or the need for meticulous tissue handling, especially in cases with significant inflammation or adhesions. These findings underscore the importance of tailored perioperative care to optimize recovery and minimize hospital stays.

In our study, while the broader literature often suggests comparable oncological outcomes between laparoscopic and open approaches for liver and biliary tumors, our findings indicate potential advantages of the open surgical method. Open surgery provides surgeons with tactile feedback and direct visualization, facilitating meticulous tissue handling and precise identification of tumor boundaries. This capability can lead to achieving wider resection margins and thorough lymph node dissection, which are critical for ensuring oncological clearance, especially in complex or extensive cases. Additionally, intraoperative ultrasonography (IOUS) emerged as a significant factor in enhancing surgical precision and survival outcomes, particularly noted before propensity score matching. IOUS aids in real-time imaging of liver anatomy and tumor characteristics, allowing for accurate lesion localization and assessment of vascular involvement, thereby supporting both open and laparoscopic procedures in achieving comparable radical resections. These insights underscore the complementary roles of open surgery's tactile advantages and IOUS's imaging capabilities in optimizing treatment outcomes for liver and biliary tumors.

Although many previous studies have demonstrated the efficacy and safety of laparoscopic surgery in iGBC patients, the vast majority of patients included in these studies were patients diagnosed with iGBC postoperatively. Our study is the only to assess the effect of COR and CLR on the survival of ID-iGBC patients. In addition to focusing on the long-term prognosis of patients, we also focused on the short-term clinical outcomes of the patients after surgery. This study performed propensity score matching on the two groups of patients, minimizing the impact of retrospective study bias on the conclusions. This study provides new evidence for clinical diagnosis and treatment. However, some limitations of this study should be considered when interpreting the results. First, it was retrospective and had inherent limitations in its design. Thus, clinical bias was inevitable. Second, due to the small sample size of the study, patients were only divided into COR and CLR subgroups, and the impact of more surgical techniques on prognosis was not studied. Third, the single-center nature of the study may lead to universal conclusions of the study subject to certain restrictions. Fourth, incomplete data collection was a challenge despite comprehensive efforts in follow-up; some patients were lost to follow-up or had missing data, which prevented us from obtaining complete information necessary for a robust analysis of DFS and recurrence. Therefore, future randomized controlled trials and large-scale multicenter prospective cohort studies are required for further verification.

## Conclusion

In conclusion, this is the only study to assess the effect of continuation of laparoscopic extensive-resection and conversion to laparotomy extensive-resection on patients’ survival. Patients with ID-iGBC in the COR group had fewer perioperative complications and better prognosis than those in the CLR group. For patients with ID-iGBC, conversion to laparotomy extensive-resection appears to be a better choice.

## Data Availability

The original contributions presented in the study are included in the article/Supplementary Material, further inquiries can be directed to the corresponding authors.
